# Carotid Sinus Massage During Head-up Tilt Testing Can Predict the Test Outcome: Implications for Its Use as a Screening Tool in Patients with Unexplained Syncope

**DOI:** 10.19102/icrm.2024.15101

**Published:** 2024-10-15

**Authors:** Atul Prakash, Julie Truong, Adeniyi Adelakun, Ravnit Singh

**Affiliations:** 1 Division of Cardiology, St. Mary’s General Hospital, Passaic, NJ, USA; 2 Division of Internal Medicine, St. Mary’s General Hospital, Passaic, NJ, USA; 3 Division of Cardiology, Saint Michael’s Medical Center, Newark, NJ, USA

**Keywords:** Carotid sinus massage, head-up tilt testing, loss of consciousness, provocative maneuvers, syncope

## Abstract

Head-up tilt testing (HUT) has been used for decades in the work-up of patients presenting with syncope and a suspected reflex etiology. Different protocols have been used with varying sensitivity and specificity. The standard protocols are relatively long, with various maneuvers employed to elicit a response and potentially abbreviate the test. The role of carotid sinus massage (CSM) as a provocative maneuver has not been well studied. The objective of this study was to assess whether CSM could predict the outcome of HUT. Fifty consecutive patients who had been referred for head-up tilt table testing were prospectively enrolled in the study. All patients underwent an identical protocol that involved provocation with CSM both initially in the supine posture and at the end of 30 min of HUT. Seventeen out of 50 (34%) patients ultimately had a positive tilt table test result. Fifteen of these 17 patients had a significant vasodepressor response (symptomatic blood pressure drop of >20 mmHg) without significant bradycardia (heart rate of <50 bpm) during the initial CSM in the supine posture. Of the 33 patients with a negative tilt table result, none had a vasodepressor response to CSM. The sensitivity of CSM in detecting a patient who would ultimately have a positive tilt table test was 88.24% (95% confidence interval [CI], 63.56%–98.54%), while the specificity was 100% (95% CI, 89.42%–100.00%). CSM performed in the supine posture at the beginning of a tilt table test was highly sensitive and specific for the outcome of the test after completion of the entire protocol. Based on these findings, CSM may obviate the need for completion of the protocol for diagnostic reasons.

## Background

Head-up tilt (HUT) table testing has been used over the last three decades to help manage patients with neurocardiogenic syncope. It was first adopted as a diagnostic test in the 1980s.^1^ Its current role is restricted to the evaluation of a mechanism of syncope when a diagnosis is unclear.^2,3^ Despite conflicting viewpoints, the test has been proven valuable in patients where the diagnosis is not clear. The test is also used to select the choice of drug therapy and the patients who may benefit from pacing. The selection of pacing has been on the basis of demonstration of asystole of ≥3 s.^4^

The emergence of implantable loop recorders (ILRs) has assumed great importance in the evaluation of syncope. These devices, however, do not address the mechanism-related questions, especially the presence of hypotension. There can also be a delay in the diagnosis due to waiting for symptom recurrence and indeed establishing a diagnosis when the syncopal event has resulted in injury.^4,5^ The ILR has also been used to select patients for pacing therapy with a diagnosis of reflex syncope in whom the HUT did not reveal any significant bradycardia.^4^

The criticism about tilt table testing has resulted from the duration of the test, reproducibility of the outcome when the test is repeated, and the sensitivity of the test.^6^

However, the sensitivity of the test significantly increases when the test duration is prolonged and provocative maneuvers and drugs are used.^7^ The drugs used are isoproterenol and nitroglycerine. These drugs increase the sensitivity of the test, albeit with some reduction in the specificity.^8^

Carotid sinus massage (CSM) is mainly used to elicit carotid sinus hypersensitivity.^9,10^ Its role in evaluating a vasodepressor response has not been extensively evaluated.

In this study, we evaluated the role of CSM in diagnosing a vasodepressor response during HUT and to establish if this had a role in predicting the outcome of the test.

## Methods

### Inclusion criteria

This prospective study included consecutive patients with symptoms of syncope and/or presyncope where the diagnosis was unclear. Some of these patients had been empirically treated with drugs for reflex syncope without improvement. Every patient seen in the office was considered for inclusion in the study.

Informed consent was obtained from the patients for the clinically indicated tilt table testing, which included CSM. Consent was also recorded to analyze the tilt table testing data. The study was reviewed by the local institutional review board.

Patients who refused to give informed consent; those with a classical history of reflex syncope; those with syncope and a history highly suggestive of carotid sinus hypersensitivity; those with significant coronary artery disease, cardiomyopathy, or congestive heart failure; those with carotid bruits (carotid Doppler was not a requirement for inclusion/exclusion); and those in whom the diagnosis was suggestive of a significant tachycardia or bradycardia were excluded.

### Statistical analysis

Statistical analyses were performed using MedCalc for Windows, version 19.4 (MedCalc Software, Ostend, Belgium). In our analysis, we treated tilt table testing as the “gold standard” for detecting reflex syncope. We then estimated the sensitivity and specificity of CSM in reference to a patient’s tilt table test result. The exact Clopper–Pearson confidence intervals (CIs) were reported for these estimates.

### Tilt testing protocol

Patients who were included underwent a tilt testing protocol. Drugs that the patients may have been empirically placed on to treat their syncope (such as β-blockers, vasodilators, and vasoconstrictors) were stopped for 72 h before the procedure. Patients underwent tilt table testing in a fasting state.

Blood pressure (BP) monitoring with the standard cuff was performed every minute. Beat-to-beat BP monitoring was also performed non-invasively. Both were done throughout and for the duration of CSM, and cuff BP monitoring was additionally performed at the end of CSM each time. Beat-to-beat BP monitoring was not used to determine the outcome of the test.

Baseline CSM was performed on the right and left carotid arteries in the supine posture. The presence of a carotid bruit was excluded by auscultation in all patients. The patient then underwent HUT at 70° for a period of 30 min. CSM was performed a second time while the patient was head up at 70°. Isoproterenol was infused at a rate of 1–3 μg/min. This was done after repeating CSM in the head-up position.

The tilt table test was terminated if the patient became syncopal and continuation of the test was deemed unsafe at any stage. Isoproterenol was also stopped when this occurred or when the patient was intolerant to it.

A positive CSM for eliciting a vasodepressor response was when the patient complained of real-life symptoms with a drop in BP by ≥20 mmHg with or without a drop in heart rate of <50 bpm.

A tilt table test was labeled as positive when the patient had symptoms identical to the real-life symptoms with a drop in BP of ≥20 mmHg. Reproducibility of the patient’s symptoms was essential in labeling the test as positive; patients who did not have this response were labeled as having had a negative tilt table test. There were six patients who were labeled as having a borderline positive test and were classified into the positive group. This was as a result of symptoms not being entirely similar to real-life symptoms but meeting the objective criteria. This was also based on an understanding that the interpretation of HUT is not an absolute positive or negative.

## Results

### Patient population

Fifty consecutive patients were included in this study; 40 were women. The mean age was 52.8 ± 3.1 years **(Supplementary Table S1)**. Thirty patients had frank syncope; the remaining had presyncope. Forty-six patients had no structural heart disease; one had an atrial septal defect repaired in July 2021, another had aortic stenosis s/p aortic valve replacement, one had a non-rheumatic mitral prolapse, and the last patient with structural heart disease had mitral regurgitation. Several patients were taking a medication that could affect BP **(Supplementary Table S2)**.

Twenty (40%) of the patients had a history of concomitant hypertension. There were 11 patients who had been treated empirically for vasodepressor syncope prior to the tilt testing by their referring physician.

**Figure 1:**
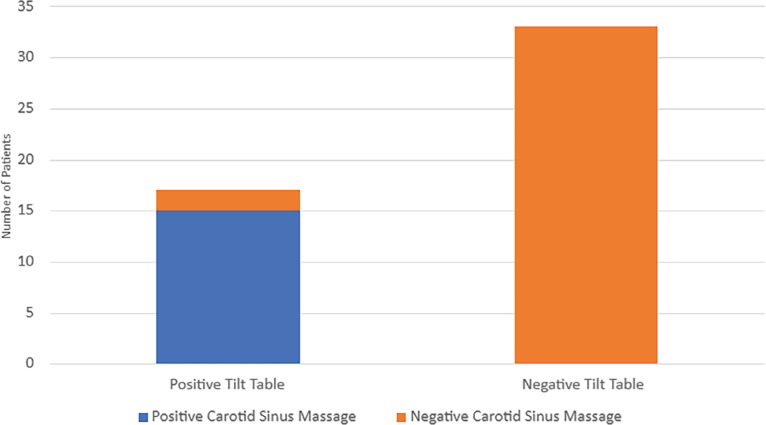
Positive and negative responses to carotid sinus massage and tilt table testing.

Seventeen out of 50 (34%) patients had a positive tilt test result for a vasodepressor response. All these 17 patients had the initial carotid massage done in which beat-to-beat BP measurements were taken every minute. Fifteen of these 17 patients had a significant vasodepressor response (symptomatic BP drop of >20 mmHg) without significant bradycardia (HR <50 bpm) during the initial CSM in the supine posture **(Supplementary Table S3 and Figure 1)**. The positive predictive value of CSM in predicting a positive tilt test was 0.884. This meant that 88.24% of the patients with a positive tilt test had a positive carotid massage. The sensitivity of CSM in detecting a patient who would ultimately have a positive tilt table test was 88.24% (95% CI, 63.56%–98.54%), while the specificity was 100% (95% CI, 89.42%–100.00%). The other two patients had a negative initial CSM.

Of the 15 patients with a positive vasodepressor response with CSM, 10 patients became positive during the head-up tilt test and 5 showed positive results during the isoproterenol infusion. These 10 patients had a significant drop in BP, which spontaneously recovered during the tilt test. This reproduced the patient’s clinical symptoms of lightheadedness.

Two of the 17 patients with an overall positive tilt test result but a negative response on CSM demonstrated a positive result only after isoproterenol infusion. Of these two patients, one did exhibit a positive response when CSM was repeated during the head-up posture after completion of 30 min of HUT.

Six of these 17 patients with an overall positive result on protocol completion had symptoms that did not entirely correlate with clinical symptoms in terms of character and/or intensity. They however met objective criteria of a positive test as defined earlier. These patients, including the two described earlier with a negative supine CSM, also required isoproterenol to yield a positive result.

Despite protocol completion, 33 out of 50 (66%) patients had a negative tilt result for a vasodepressor response. All 33 patients had no drop in BP during the initial CSM and no significant bradycardia. They all underwent the head-up tilt test for 20 min with no response. A second carotid massage was done, which produced no symptoms. Lastly, isoproterenol was infused up to 3 μg and still produced no neurocardiogenic response. Overall, the tests were negative for these 33 patients. The negative predictive value was 1, indicating that 100% of the patients with a negative tilt test had a negative carotid massage.

## Discussion

Tilt table testing has been the cornerstone for the diagnosis of neurocardiogenic syncope.^1,11^ However, multiple factors have led to its relegation to a class Il(a) indication and underuse in electrophysiology laboratories. The criticism has been a lack of reproducibility and the time consumed and perhaps the financial implications. The advent of implantable loop monitors also facilitated the underuse of the test. The proponents of the test state that the test still has a role in selecting a treatment regimen drug or pacing in such patients.^11^ Different protocols have been used, which are time-consuming. The major criticism has been that this is a clinical diagnosis and that the test should not be used to diagnose this condition. This study provides an alternative diagnostic tool that may be implemented at bedside or in the office in most patients with suspected neurocardiogenic syncope. The test may constitute a method where at least patients with a positive response may undergo the full test to aid in determining the therapy. The findings certainly do not suggest not doing HUT but rather suggest CSM being used as a screening tool.

Our population in this study is different from that in the existing literature.^1^ The patients’ history was not clearly diagnostic of a reflex etiology.^2^ This may reflect the low incidence of positive results. Patients with abrupt-onset syncope and loss of consciousness highly suggestive of carotid sinus hypersensitivity were excluded. This may explain why bradycardia was not the dominant finding on test completion. It is not clear whether the mechanism in these patients is a milder version of carotid sinus hypersensitivity or there remains a different mechanism. The use of CSM was evaluated in both studies; however, the populations of these studies were different. In a large study of over 1000 patients, CSM was used in an elderly population different from ours where the findings of bradycardia and hypotension were seen. The test was deemed safe in this population of patients.^11^

The protocols used and recommended for tilt table testing have ranged from 20–45 min and up to 2 h,^12^ with head-up angles ranging from 40° to 60°. In a comprehensive meta-analysis of 55 studies evaluating symptomatic and asymptomatic patients, there were 4361 patients with unexplained syncope and 1791 controls. The study reveals an excellent ability to differentiate symptomatic patients from overall controls, with a 95% CI of 0.81–0.87 and an area under the receiver operating curve of 0.84.

Tilt protocols that included nitroglycerin had the highest diagnostic odds ratio and greatest sensitivity (66%; 95% CI, 60%–72%).^9^ Isoproterenol has similar results with increased sensitivity of the test in eliciting a vasodepressor response. Currently, the availability of isoproterenol is a deterrent in the United States because of its significantly high costs.

CSM during tilt table testing has mainly been used to elicit episodes in patients with carotid sinus hypersensitivity.^9^ There are limited data on the use of this maneuver to elicit a vasodepressor response, especially in the younger population. In a large study of patients over the age of 40 years, the overall diagnostic yield was 61%, which was mainly for bradycardia. The conclusion was that CSM was poor in eliciting a hypotensive response.^10^

The mechanism of carotid sinus hypersensitivity merits discussion. The carotid sinus harbors baroreceptors, which are innervated by the glossopharyngeal nerve. By virtue of the connection of the nerve with the solitary nucleus in the medulla, CSM results in vagal stimulation. On carotid sinus stimulation, there is either a predominant bradycardia response, a pure vasodepressor response, or a mixed response. A pause of >3 s is defined as a hypersensitive carotid sinus.

In our study, we have identified excellent sensitivity in patients who eventually had a negative HUT outcome. Of course, this is on the assumption that a complete tilt test has 100% sensitivity to diagnose vasodepressor syncope. The implications of the results are that, in patients with syncope with an unclear etiology, a bedside CSM may exclude a vasodepressor etiology. Based on these findings, a negative bedside response may obviate the need to do the full test in the laboratory. If, however, the clinical symptom complex is highly suggestive of a reflex etiology, one may still consider the test. A patient with a positive response on CSM may be considered for the full HUT mainly to select therapy pacing or drugs and even the type of drug. The type of response and the presence and/or absence of significant bradycardia may have a role in the choice of the drug and pacing.

There are emerging newer modalities of treatment for reflex syncope, including cardioneuroablation and different algorithms in implanted pacemakers. The aims of this analysis are merely to identify a screening tool and to perform HUT in patients to understand the mechanism and select the appropriate treatment modality.

### Limitations

The role of CSM has been compared with the eventual outcome of the complete tilt test. The sensitivity and specificity have not been calculated with respect to a clinical disease status, rather in relation to another, more cumbersome test. The population in the study included patients in whom the symptoms were not diagnostic of a reflex. This also explains the low incidence of a positive result on completion of the test. Patients with highly likely carotid sinus hypersensitivity based on history were not included. The episodes of syncope and presyncope were not numerically quantified, as the intention was to evaluate the role of CSM and not necessarily to determine the outcome. We also recognize that, with a small sample size as such and the study being a single-center study, there is a need to conduct a larger investigation to derive definite conclusions.

## Figures and Tables

**Supplementary Table S1:** Patients Categorized by Age and Sex

Age (years)	15–25	24–44	45–64	65–85
Female sex	2	12	17	7
Male sex	1	1	1	6

**Supplementary Table S2:** Medications Capable of Altering Blood Pressure That Patients Were Taking Before Their Tilt Table Study

Medication	Number (%) of Patients
Droxidopa	1 (2)
Fludrocortisone	2 (4)
Midodrine	9 (18)
Diuretic	4 (8)
ACE inhibitor or angiotensin receptor blocker	6 (12)
Calcium channel blocker	3 (6)
β-Blocker	10 (20)
Tamsulosin	1 (2)

*Abbreviations:* ACE, angiotensin-converting enzyme; BP, blood pressure.

**Supplementary Table S3:** Contingency Table of Results of Tilt Table and Carotid Sinus Massage Testing in All 50 Patients

		CSM Result	
		Positive	Negative
**Tilt table test result**	**Positive**	15	2
	**Negative**	0	33

*Abbreviation:* CSM, carotid sinus massage.
